# Gender differences in the prevalence and clinical correlates of thyroid dysfunction in patients with first-episode and drug-naïve major depressive disorder with comorbid suicide attempts: a large cross-sectional study

**DOI:** 10.1186/s12888-023-05089-w

**Published:** 2023-08-18

**Authors:** Xiao Huang, Yuan Sun, Anshi Wu, Xiang-Yang Zhang

**Affiliations:** 1 Department of Anesthesiology, Beijing Chao-Yang Hospital, Capital Medical University, Beijing, China; 2 Department of Pharmacy, Peking University Third Hospital, Beijing, China; 3 CAS Key Laboratory of Mental Health, Institute of Psychology, Beijing, China; 4 Department of Psychology, University of Chinese Academy of Sciences, 16 Lincui Road, Chaoyang District, Beijing, 100101 China

**Keywords:** Gender, Abnormal thyroid function, Major depressive disorder, Suicide

## Abstract

**Background:**

Gender differences in patients with major depressive disorder (MDD) are commonly reported; however, gender differences in first-episode and drug-naïve (FEDN) MDD patients with comorbid suicide attempts have not been reported. This study aimed to examine potential gender differences in the prevalence and clinical correlates of comorbid abnormal thyroid function (ATF) in FEDN MDD patients with comorbid suicide attempts.

**Methods:**

A cross-sectional study of 1718 FEDN MDD patients was conducted. The demographic and clinical data were collected. The Hamilton Depression Scale (HAMD), the Hamilton Anxiety Scale (HAMA) and Positive and Negative Syndrome Scale (PANSS) were used to assess depression, anxiety and psychotic symptoms, respectively. Thyroid function parameters and blood glucose levels were measured.

**Results:**

There was no gender difference in the prevalence of ATF between male (78.6%, 88/112) and female MDD patients (74.8%, 175/234) with comorbid suicide attempts. In the male and female subgroups, duration of disease, HAMD score, HAMA score, anti-thyroglobulin (TgAb), thyroid peroxidases antibody (TPOAb), diastolic blood pressure (DBP), systolic blood pressure (SBP), glucose level and the rate of psychotic symptoms were higher in patients with ATF than those without ATF in MDD with comorbid suicide attempt (all *P* < 0.05). There was a gender main effect only on SBP (*F* = 7.35, *P* = 0.007). Furthermore, binary logistic regression analysis showed that HAMD score, DBP and glucose levels were independently with ATF in both male and female MDD patients with comorbid suicide attempts. However, anxiety symptoms, psychotic symptoms and TPOAb levels were significantly associated with ATF only in female MDD patients with comorbid suicide attempts.

**Conclusion:**

Our study showed no gender differences in the prevalence of ATF in FEDN MDD patients with comorbid suicide attempts. Depression, DBP and glucose levels were associated with ATF in both male and female MDD patients with comorbid suicide attempts, whereas anxiety, psychotic symptoms and TPOAb level were correlated with ATF only in female MDD patients with suicide attempts.

## Introduction

Major depressive disorder (MDD) is a serious public health problem with a significant disease burden worldwide [1]. MDD has many potential risk factors, including chronic disease, metabolic syndrome, smoking, frequent alcohol consumption, and low physical activity [2]. The prevalence of MDD is approximately twice as high in women as in men [3]. Most risk factors have a greater impact on MDD risk in women than in men, and cross-gender risk factors typically have a deeper effect on potential recurrent MDD than on the first episode of MDD [4]. Although overall gender differences in prevalence of MDD have been well explored, gender differences in other aspects have received limited attention historically.

Suicide is prevalent in MDD [5–7]. The results of a study conducted in Brazil showed that the cumulative prevalence of suicide attempts in MDD patients was 10.1%, and that people who were previously at risk for suicide were more likely to attempt suicide [8]. Another study of depressed patients in China showed that the prevalence of suicidal ideation among depressed patients was 53.4%, and that women were more likely to have suicidal ideation than men [9].

The relationship between thyroid dysfunction and mood disorders has been extensively studied over the past few decades. Both deficiency and excess of thyroid hormones may be accompanied by various neuropsychiatric symptoms, including depression. Patients with MDD have significantly lower free triiodothyronine (FT3), free thyroxine (FT4) and thyrotropin (TSH) compared to healthy controls [10]. Even variations in thyroid function within the normal range have been associated with the risk of MDD [11]. It has been proposed that MMD is associated with changes in the hypothalamic-pituitary-thyroid (HPT) axis and hypercortisolism [12]. A previous study found that suicide attempts and psychotic symptoms were associated with MDD patients with severe subclinical hypothyroidism [13].

Thyroid disease is a very prevalent disorder that influences the entire body, both in cognitive function and mental health. Therefore, thyroid disorders are involved in a variety of neuropsychiatric disorders. Patients with suicidal behavior demonstrate significantly reduced mean FT3 and TT4 levels compared to patients without suicidal behavior [14]. Liu et al. concluded that patients with abnormal TSH must be thoroughly screened for suicidal ideation [15]. Heiberg et al. found that Hashimoto’s thyroiditis may play an important role in the pathophysiological underlying mechanisms of suicidal behavior [16]. The pathophysiology of suicidal behavior may be related to simultaneous alterations in the dopaminergic (DA) and hypothalamic-pituitary-thyroid systems [17]. In view of the above studies, suicide and ATF are closely related with each other and have a complex impact on MDD. Therefore, comorbid ATF in suicide attempts has important research value.

Gender differences play an important role in depression comorbidity and influence the course of the disease. MDD comorbid suicide attempts are common in both men and women, but suicide attempts in female MDD patients are reported at a higher rate compared to male MDD patients [18]. Previous studies compared gender difference in the thyroid dysfunction between male and female patients with psychiatric disorders are still controversial. For example, data from a Korean National Health and Nutrition Examination Survey found that gender may play an important role in the relationship between TSH and depressive symptoms. The highest TSH tertile in men was 1.92 times more likely to have depressive symptoms than the lowest TSH tertile, while the highest TSH tertile in women was associated with an approximately 35% lower prevalence of depressive symptoms in women [19]. Varella et al. showed that low TSH concentration were linked with incident depression in women but not in men [20]. The prevalence of thyroid disorders is much higher in women than in men with bipolar disorder and increases with age. The most common abnormality is subclinical hypothyroidism, which occurs in up to 20% of postmenopausal women [21].

Gender differences are important in psychiatric disorders, which may contribute to our understanding of the mechanisms underlying the onset and maintenance of mood disorders in men and women. However, gender differences in ATF among MDD patients with comorbid suicide attempts still receive limited attention. There is an urgent need to develop gender-specific interventions to improve the mental health of this specific Chinese population. Therefore, we investigated gender differences in ATF between first-episode and drug-naïve (FEDN) MDD patients with suicide attempts in a large sample of Chinese Han population (*N* = 1718). The purpose of this study was to explore (1) gender differences in the prevalence of ATF among MDD patients with comorbid suicide attempts; and (2) gender differences in demographic characteristics and clinical factors associated with ATF in FEDN MDD patients with comorbid suicidal attempts. We hypothesized that there would be gender differences in the prevalence and clinical correlates of ATF in FEDN MDD patients with comorbid suicide attempts.

## Methods

### Procedure and sample

This cross-sectional study was conducted in the First Hospital of Shanxi Medical University in 2015–2017. We recruited FEDN MDD outpatients. Inclusion criteria were as follows: (1) age 18–60 years; (2) first-episode depressive symptoms, and no any previous medication, including antidepressant or antipsychotic drugs; (3) diagnosis of MDD by two psychiatrists, using the Structured Clinical interview for Diagnostic and Statistical Manual of Mental Disorders, 4th edition (DSM-IV); and (4) 17-item HAMD total score ≥ 24. Exclusion criteria included: (1) pregnancy or breastfeeding (*n* = 10); (2) substance use disorder (*n* = 9) except for nicotine; (3) severe personality disorder (*n* = 15); (4) severe physical illness (*n* = 9); (5) refused to participate in the trial (*n* = 21); (7) unable to be interviewed because of acute clinical conditions (*n* = 5) and (8) other unknown reasons (*n* = 9). No minor (age less than 16 years) participants were involved in this study. The minimum years of the education that participants have received was 9. No uneducated participants were involved in this study.

Altogether, 1796 individuals were screened. Seventy-eight patients were ruled out for the following reasons: (1) serious physical illness and personality disorder (*n* = 24); (2) drug abuse and dependence (*n* = 9); (3) female patients who were pregnant or nursing (*n* = 10); (4) who were not available for interview due to an acute clinical condition (*n* = 5), (5) Reluctance to give a written consent form (*n* = 21) and other unspecified reasons (*n* = 9). There were 1718 outpatients in total included from the Department of Psychiatry of this hospital finally.

This study was approved by the Institutional Review Board (IRB) of First Hospital, Shanxi Medical University. All patients were informed of the trial procedure and signed an informed consent form before participating in this study.

### Demographics, clinical and biomarker measurements

We collected socio-demographic and clinical data including age, sex, body mass index (BMI), age at onset, duration of disease, education level and marital status through a questionnaire. Other information was collected from medical records and other data sources.

Clinical psychiatric evaluation including The 14-item Hamilton Anxiety Rating Scale (HAMA) [22], The 17-item Hamilton Depression Rating Scale (HAMD) [23] and Positive and Negative Syndrome Scale (PANSS) [24], which are usually used in patients with anxiety, depression and psychotic symptoms, respectively. The HAMA consists of 14 items and two factor types Somatic anxiety (items 7, 8, 9, 10, 11, 12, and 13) and mental anxiety (items 1, 2, 3, 4, 5, 6, and 14), using a 0–4 scale depending on the severity of each symptom. The HAMD consists of 17 items, the majority of which are rated on a 0–4 scale according to the severity of each symptom, while the other items (insomnia, gastrointestinal symptoms, general symptoms, weight loss, and insight) are rated on a 0–2 scale. Occurrence and severity of depression was evaluated by HAMD rating cut-offs: ≦ 7 = no depressed at all, ≧ 8 = depressed, ≦ 17 = mild to moderate depressive states, ≥ 24 = severe depression. In this study, 24 was the cut-off value to divide patients into groups with and without serious depressive symptoms [25]. Patients with HAMA score greater than 18 were defined as having severe anxiety symptoms [26]. Patients with a positive PANSS subscale score of more than 15 were defined as having psychotic symptoms [27].

These scales were evaluated by two psychiatrists with at least 5 years of clinical experience. Two psychiatrists received prior professional training. For the three clinical evaluation scales mentioned above, the internal consistency coefficients of the HAMD, HAMA and PANSS total scores were 0.85, 0.84 and 0.82, respectively.

Fasting blood samples were collected from all patients at 6–8 am before participants underwent any medication. Samples were tested before 11 a.m. of the same day as the clinical data were collected to test the following biochemical parameters: FT3, FT4, TSH, anti-thyroglobulin (TgAb), thyroid peroxidases antibody (TPOAb), and fasting glucose. Thyroid hormones (TSH, TPOAb, TgAb, FT3, and FT4) were assessed by a Roche C6000 Electrochemiluminescence Immunoassay Analyzer (Roche Diagnostics, Indianapolis, IN, USA), while glucose were measured by a Cobas E610 (Roche, Basel, Switzerland) in the laboratory of Shanxi Medical University. The blood pressure, height, and weight were measured by trained nurses. Body mass index (BMI) was obtained with the equation: BMI = Weight (kg)/Height (m)^2^. In the present study, ATF was defined as TSH > 4.2 mIU/L [28, 29].

### Statistical analysis

The Kolmogorov-Smirnov one-sample test was used to examine the distribution of quantitative variables. For descriptive analysis, qualitative variables were expressed as numbers (percentages), quantitative variables with normal distribution were expressed as mean ± SD, and quantitative variables with non-normal distribution were tested by Mann-Whitney U test. Next, categorical data were compared male and female suicide attempters using chi-square tests and continuous variables using ANOVA (one-way analysis of variance). Bonferroni correction was used for each test to accommodate multiple testing. Then to compare gender differences in ATF among MDD patients with suicide attempts, we used a 2 × 2 ANOVA considering diagnosis (2 levels: with and without ATF) and gender (2 levels: male and female). The main effects of gender and diagnosis, and the gender* diagnosis interaction were examined. Binary logistic regression analysis (Backward: Wald) was then used to explore the factors associated with ATF in male and female MDD patients with suicide attempts, respectively. We used variance inflation factors (VIF) to assess multicollinearity between independent variables. Those with VIF greater than 2.5 should be excluded from the model. Finally, significant variables from the logistic regression were included in areas under Receiver Operating Characteristics (AUC ROC) to determine their ability to distinguish between patients with and without ATF in male and female MDD patients, respectively.

All *P* values were two-tailed with a significance level of ≤ 0.05. Statistical analyses were performed using SPSS 25 (SPSS, Inc., Chicago, IL).

## Results

### Gender differences in demographic and clinical parameters of FEND MDD patients with comorbid suicide attempts

There was no gender difference in the prevalence of comorbid suicide attempts in MDD patients (male: 19.1%, female: 20.7%; *F* = 0.56, *P* = 0.453).

Among male MDD patients with and without suicide attempts, the prevalence of ATF was 78.6% (88/112) and 56.7% (270/476), respectively (*P* < 0.001, OR = 2.80, 95% CI = 1.72–4.55). Among female MDD patients with and without suicide attempts, the prevalence of ATF was 74.8% (175/234) and 57.0% (511/896), respectively (*P* < 0.001, OR = 2.24, 95% CI = 95%1.62–3.09). However, there was no gender difference in the prevalence of ATF between male (78.6%, 88/112) and female (74.8%, 175/234) MDD patients with comorbid suicide attempts (*F* = 0.41, *P* = 0.524). Table 1 shows no gender differences in demographic and clinical parameters among FEND MDD patients with suicide attempts.

**Table 1:** Gender difference: socio-demographic and clinical characteristic among FEDN patients with MDD with comorbid suicide attemtps

	**MDD with suicide attemtps**		**F**	***P***
	**Male (112)**	**Female (234)**		
**Age (years)**	34.6 (12.7)	36.9 (12.2)	2.529	0.113
**Age of onset (years)**	34.4 (12.6)	36.6 (12.1)	2.59	0.108
**Duraton of disease (months)**	7.0 (5.2)	7.0 (4.8)	0.002	0.969
**BMI**	24.0 (2.7)	24.5 (2.1)	2.544	0.112
**Education, n (%)**			6.754	0.08
**1**	24 (21.4)	76 (32.5)		
**2**	49 (43.8)	92 (39.3)		
**3**	33 (29.5)	48 (20.5)		
**4**	6 (5.4)	18 (7.7)		
**Married, n (%)**	82 (73.2)	169 (72.2)	0.004	0.847
**HAMD**	32.1 (3.1)	32.3 (2.8)	0.45	0.503
**HAMA**	23.3 (3.4)	23.8 (3.6)	1.445	0.23
**PANSS**	11.0 (6.1)	11.7 (6.7)	1.046	0.307
**Severe anxiety, n (%)**	32 (28.6)	79 (33.8)	0.713	0.398
**Exhibiting psychotic symptoms, n (%)**	25 (22.3)	63 (26.9)	0.846	0.358
**ATF**	88 (78.6)	175 (74.8)	0.406	0.524

Data expressed as mean ± SD or percentage

Education degree: 1, middle school; 2, high school; 3, college; 4, graduate

*BMI* Body mass index, *HAMD* Hamilton Rating Scale for Depression, *HAMA* Hamilton Anxiety Scale, *PANSS* Positive and Negative Syndrome Scale, *ATF* Abnormal thyroid function

### Gender differences in clinical characteristics and biochemical parameters between the ATF and non-ATF subgroups in MDD patients with suicide attempts

As shown in Table 2, ANOVA was performed to examine the interaction between ATF and gender. A two-way ANOVA showed that ATF had a significant effect on disease duration (*F* = 5.17, *P* = 0.024), HAMA score (*F* = 7.02, *P* = 0.008), HAMD score (*F* = 39. 03, *P* < 0.001), PANSS positive subscale score (*F* = 24.53, *P* < 0.001), psychotic symptoms (*F* = 17.84, *P* < 0.001), DBP (*F* = 25.788, *P* < 0. 001), systolic blood pressure (SBP) (*F* = 53.458, *P* < 0.001), glucose levels (*F* = 68, *P* < 0.001), TPOAb (*F* = 30.92, *P* < 0.001), and TgAb (*F* = 27.35, *P* < 0.001). However, disease duration and HAMA score did not survive after Bonferroni correction. There was a gender main effect only on SBP (*F* = 7.35, *P* = 0.007). In addition, there were no significant ATF*gender interaction on any of the variables (all *P* > 0.05) (Table 2).

**Table 2:** Demographic and clinical characteristics between MDD patients with comorbid suicide attempt with and without ATF, grouped by gender

**Characteristic**	**MDD comorbid suicide attempt**						
	**With ATF**		**Without ATF**		**ATF F (*P*-value)**	**Gender F (*P*-value)**	**ATF × Gender F (*P*-value)**
	**Male (*n* = 88)**	**Female (*n* = 175)**	**Male (*n* = 24)**	**Female (*n* = 59)**			
**Age (years)**	34.66 (12.69)	36.85 (12.23)	34.38 (12.89)	36.86 (12.04)	0.00 (0.984)	2.52 (0.113)	0.01 (0.929)
**Age of onset (years)**	34.43 (12.55)	36.59 (12.11)	34.08 (13.03)	36.71 (12.01)	0.00 (0.956)	2.57 (0.110)	0.02 (0.889)
**BMI**	24.13 (2.93)	24.58 (2.13)	23.67 (1.53)	24.12 (2.10)	2.28 (0.132)	2.76 (0.098)	0.00 (0.988)
**Married, n (%)**	62 (70.45)	126 (72)	20 (83.33)	43 (72.88)	0.42 (0.519)	0.00 (0.948)	
**Education, n (%)**							
**1**	20 (22.73)	55 (31.43)	4 (16.67)	21 (35.59)	0.22 (0.974)	6.75 (0.08)	
**2**	38 (43.18)	71 (40.57)	11 (45.83)	21 (35.59)			
**3**	25 (28.41)	36 (20.57)	8 (33.33)	12 (20.34)			
**4**	5 (5.68)	13 (7.43)	1 (4.17)	5 (8.47)			
**Duration of disease (months)**	7.01 (5.16)	7.42 (5.01)	6.81 (5.29)	5.52 (3.63)	5.17 (0.024)	0.00 (.956)	1.64 (0.201)
**HAMA**	23.48 (3.52)	24.07 (3.66)	22.46 (2.86)	22.80 (3.28)	7.021 (0.008)	1.75 (0.187)	0.070 (0.794)
**HAMD**	32.67 (2.94)	32.8 (2.47)	29.96 (2.85)	30.86 (3.13)	39.03 (< 0.001)	0.94 (0.334)	1.06 (0.303)
**PANSS**	11.58 (6.65)	12.84 (6.94)	8.71 (3.45)	8.424 (4.34)	24.53 (< 0.001)	1.60 (0.207)	0.80 (0.371)
**Severe anxiety, n (%)**	26 (29.55)	65 (37.14)	6 (25.00)	14 (23.73)	2.73 (0.098)	0.71 (0.398)	
**Exhibiting psychotic symptoms, n (%)**	23 (26.14)	59 (33.71)	2 (8.33)	4 (6.78)	17.84 (< 0.001)	0.62 (0.431)	
**DBP**	78.97 (8.39)	80.07 (7.19)	73.917 (6.89)	75.37 (6.82)	25.79 (< 0.001)	1.93 (0.166)	0.03 (0.865)
**SBP**	124.43 (11.16)	128.06 (10.64)	114.63 (8.80)	117.48 (12.90)	53.46 (< 0.001)	7.35 (0.007)	0.07 (0.798)
**Glucose concentration (mmol/L)**	5.73 (0.64)	5.77 (0.72)	5.19 (0.66)	4.99 (0.61)	68.0 (< 0.001)	0.05 (0.816)	1.58 (0.210)
**Log2(TPOAb),IU/Ml**	6.05 (2.18)	5.83 (2.20)	4.38 (1.38)	4.49 (1.62)	30.92 (< 0.001)	0.38 (0.516)	0.33 (0.564)
**Log2(TgAb),IU/Ml**	5.84 (1.93)	6.06 (2.01)	4.77 (1.56)	4.75 (1.32)	27.35 (< 0.001)	0.59 (0.442)	0.20 (0.657)
**FT3,pg/mL**	4.93 (0.75)	4.96 (0.69)	4.70 (0.69)	4.84 (0.79)	2.56 (0.111)	0.48 (0.487)	0.28 (0.595)
**FT4ng/dL**	16.88 (3.1)	16.57 (3.09)	16.52 (3.44)	16.76 (3.21)	0.00 (0.962)	0.27 (0.606)	0.41 (0.521)

Data expressed as mean ± SD, median (interquartile range), or percentage

Education degree: 1, middle school; 2, high school; 3, college; 4, graduate

*ATF* Abnormal thyroid function, *HAMD* Hamilton Rating Scale for Depression, *HAMA* Hamilton Anxiety Scale, *PANSS* Positive and Negative Syndrome Scale, *BMI* Body mass index, *DBP* Diastolic blood pressure, *SBP* Diastolic blood pressure, *TgAb* Anti-thyroglobulin, *TPOAb* Thyroid peroxidases antibody, *FT3* Free triiodothyronine, *FT4* Free thyroxine

### Logistic regression of risk factors for ATF among male MDD patients with suicide attempts

**Fig. 1:**
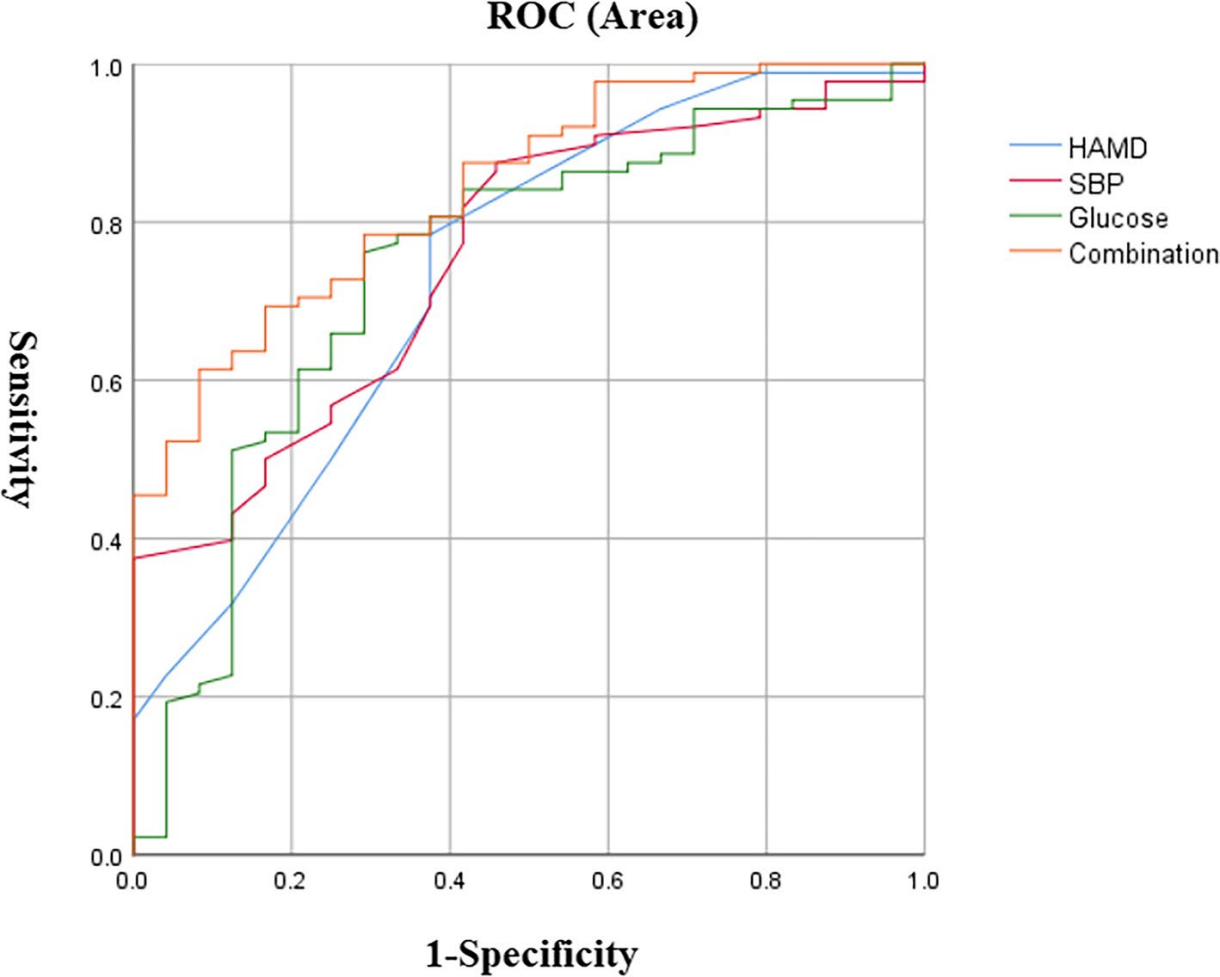
The discriminatory capacity of related factors for distinguishing between male patients with and without ATF in MDD comorbid with suicide attempt. The area under the curve of HAMD score, SBP, glucose level and the combination of these three factors were 0.735, 0.756, 0.743 and 0.842, respectively. ROC: receiver operating characteristic. ATF: abnormal thyroid function. HAMD: Hamilton Rating Scale for Depression. SBP: systolic blood pressure

Binary logistic regression showed that factors associated with ATF in male MDD patients with suicide attempts included: HAMD score (OR = 1.35, 95% CI: 1.08–1.69, *P* = 0.009), SBP (OR = 1.08, 95% CI: 1.02–1.14, *P* = 0.01) and blood glucose levels (OR = 2.73, 95% CI. 1.05–7.06, *P* = 0.039) (Table 3). Furthermore, AUC ROC showed the following values for each risk factor: 0.735 for HAMD, 0.756 for SBP and 0.743 for glucose. We then combined these parameters and found an AUC value of 0.842 to distinguish between ATF and normal patients (*P* < 0.001, 95% CI = 0.76–0.92) (Fig. 1).

**Table 3:** Binary logistic regression analyses of determinants of ATF in male FEND patients with comorbid suicide attempt

	**B**	**W**	**D**	***P***	**OR**	**95%CI Lower**	**95%CI Upper**
**HAMD**	0.23	6.80	1	0.009	1.35	1.08	1.69
**SBP**	0.08	6.65	1	0.010	1.08	1.02	1.14
**Glucose concentration**	1.00	4.26	1	0.039	2.73	1.05	7.06

*ATF* Abnormal thyroid function, *HAMD* Hamilton Rating Scale for Depression, *SBP* Diastolic blood pressure

### Logistic regression of risk factors for ATF among female MDD patients with suicide attempts

**Fig. 2:**
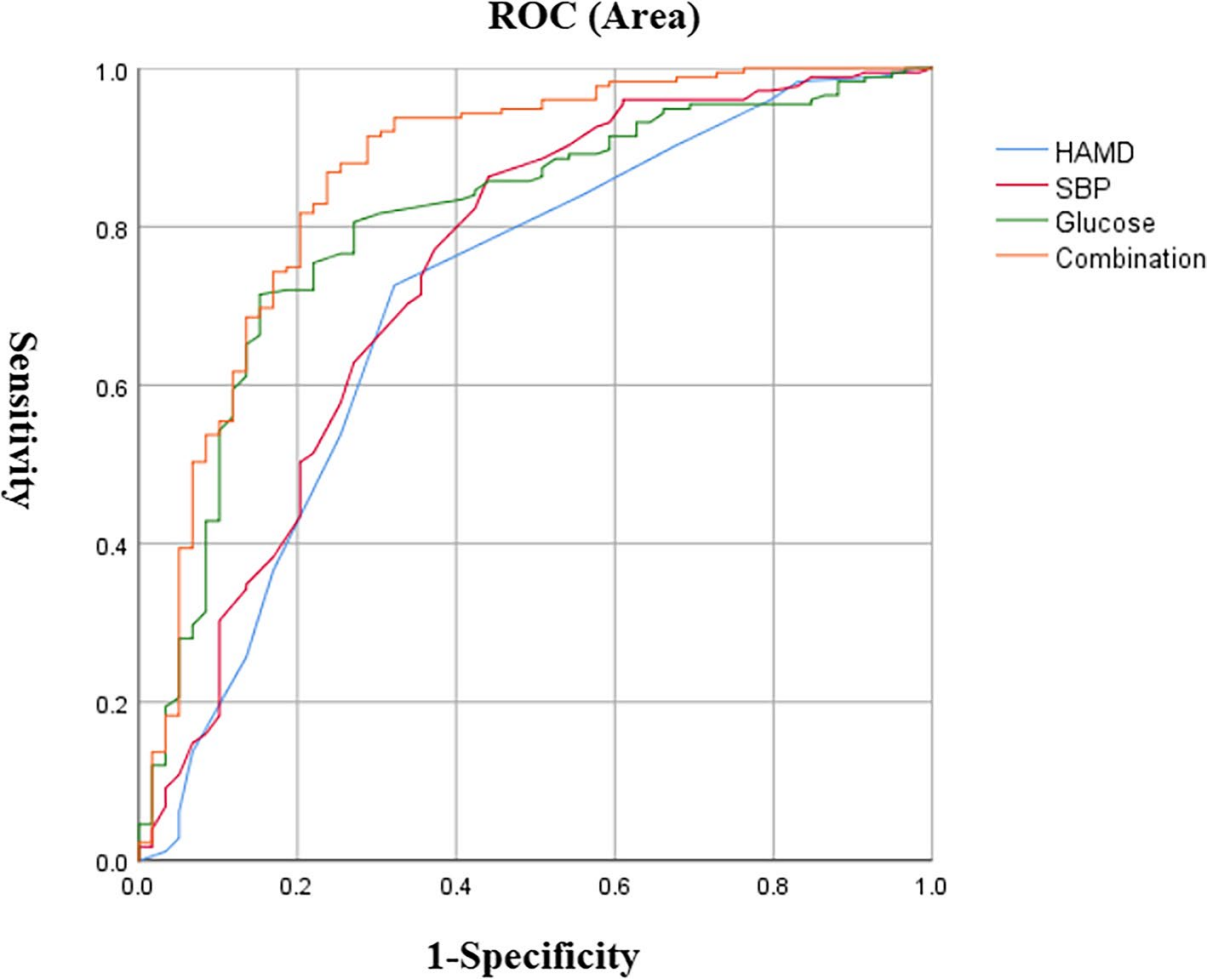
The discriminatory capacity of related factors for distinguishing between female patients with and without ATF in MDD comorbid with suicide attempt. The area under the curve of HAMD score, SBP, glucose level and the combination of these three factors were 0.708, 0.743, 0.806 and 0.864, respectively. ROC: receiver operating characteristic. ATF: abnormal thyroid function. HAMD: Hamilton Rating Scale for Depression. SBP: systolic blood pressure

Binary logistic regression showed that factors associated with ATF in female MDD patients with suicide attempts included: HAMD score (OR = 1.29, 95% CI: 1.10–1.52, *P* = 0.002), anxiety (OR = 3.47, 95% CI: 1.12–10.75, *P* = 0.031), SBP (OR = 1.07, 95% CI: 1.03–1.12, *P* < 0.001), psychotic symptoms (OR = 5.46, 95% CI: 1.21–24.71, *P* = 0.028), TPOAB level (OR = 1.01, 95% CI: 1.00–1.01, *P* = 0.005) and blood glucose levels (OR = 5.86, 95% CI: 2.94–11.69, *P* < 0.001). Furthermore, AUC ROC showed the following values for each risk factor: 0.708 for HAMD, 0.743 for SBP, 0.806 for glucose, 0.678 for TPOAB, 0.635 for psychotic symptoms, and 0.567 for anxiety. Finally, when we combined parameters with an AUC value ≥ 0.7, we found that the combination of HAMD score, SBP, and glucose had a higher AUC value of 0.84 to distinguish between ATF and non-ATF patients (*P* < 0.001, 95%CI = 0.80–0.93) (Table 4 and Fig. 2).

**Table 4:** Binary logistic regression analyses of determinants of ATF in female FEND patients with comorbid suicide attempt

	**B**	**W**	**D**	***P***	**OR**	**95%CI Lower**	**95%CI Upper**
**HAMD**	0.25	9.43	1	0.002	1.29	1.10	1.52
**Severe anxiety**	1.25	0.58	1	0.031	3.47	1.12	10.75
**SBP**	0.07	12.74	1	< 0.001	1.07	1.03	1.12
**Exhibiting psychotic symptoms**	1.70	4.85	1	0.028	5.46	1.21	24.71
**TPOAb**	0.01	7.75	1	0.005	1.01	1.00	1.01
**Glucose concentration**	1.77	25.15	1	< 0.001	5.86	2.94	11.69

*ATF* Abnormal thyroid function, *HAMD* Hamilton Rating Scale for Depression, *SBP* Systolic blood pressure, *TPOAb* Thyroid peroxidases antibody

## Discussion

To our knowledge, this is the first large clinical study to examine sex differences in ATF in FEDN MDD patients with comorbid suicide attempts. Our findings included: (1) the prevalence of ATF was high in both sexes, up to 76.0%; (2) there was no gender difference in the prevalence of ATF in MDD patients with suicide attempts; and (3) there were gender differences in risk factors for ATF in MDD patients with suicide attempts.

Our study found no difference in the prevalence of ATF between male and female MDD patients with comorbid suicide attempts. A large study of depressed inpatients by Zhao et al. found that the prevalence of subclinical hypothyroidism was approximately twice as high in female depressed inpatients as in male depressed inpatients [30]. Also, another large study by Engum et al. showed that in individuals with normal TSH and no known thyroid disease, the prevalence of TPOAb was 14.2% in women and 4.3% in men [31]. They reported that among participants with biochemical thyroid dysfunction, the prevalence of TPOAb was 59.0% in females and 38.9% in males. In the present study, the prevalence of ATF in MDD patients with comorbid suicide attempts was higher in both male and female patients than in previous studies. In contrast, no gender difference in ATF was found in the present study. This difference may be due to the different diagnostic criteria in the present study and previous studies. In the present study, we included only patients with severe MDD. On the other hand, the included patients were MDD patients with suicide attempts, therefore, suicide attempts may be an important factor influencing the gender difference in ATF.

In the present study, women reported higher SBP in ATF subgroup in MDD patients with comorbid suicide. Consistent with our findings, Duan et al. found a significantly higher incidence of hypertension in the subclinical hypothyroidism group than in the non- subclinical hypothyroidism group only in female MDD patients, while no significant difference was found between the two groups in male subjects, suggesting that SCH was an independent predictor of elevated SBP and pulse pressure only in women [32]. However, Langén et al. demonstrated that TSH was negatively associated with 11-year changes in blood pressure in men, but not in in women [33]. Some studies found no difference in systolic BP between male and female ATF patients. For example, Gu et al. found a positive association between FT3 or FT4 and elevated blood pressure in adults with normal thyroid function, but no significant relationship between TSH and elevated blood pressure [34]. These differences may be due to the fact that our sample was FEND MDD patients with comorbid suicide attempts, whereas the samples of other studies were only MDD patients or depressed patients. Therefore, our results need to be interpreted with caution.

Another major finding of this study was that there were gender differences in the clinical variables associated with ATF in MDD patients with suicide attempts. In male patients, three clinical variables and metabolic indicators-HAMD score, SBP, and blood glucose levels-significantly predicted ATF in MDD patients with suicide attempts, whereas in anxiety, SBP, psychotic symptoms, TPOAb, and glucose levels significantly predicted ATF in MDD patients with suicide attempts. Interestingly, similar results have been observed in other recent studies, showing that depressed patients with comorbid anxiety were more likely to have a higher risk of suicide attempts and psychotic features [35, 36]. Mowla et al. showed that depressed patients with hypothyroidism had more anxiety symptoms [37]. Sigman et al. found that patients with autoimmune thyroiditis were at increased risk of developing symptoms of depression and anxiety or being diagnosed with depression and anxiety [38]. Compared with healthy controls, female adolescents meeting nonsuicidal self-injury criteria exhibited altered HPT axis function due to a reduced fT3/fT4 ratio [39]. Steiner et al. indicated that MDD patients who were thyroid antibody positive had higher HAMD-21 score [40]. It is well known that there are gender differences in depressive symptoms and psychotic symptoms in patients with depression. Previous study has consistently found that coexisting depressive symptoms and psychotic features are more common in women than in men [41]. Stanton et al. showed that women reported significantly higher levels of psychotic-like experiences and associated distress than men [41]. In addition to steroid hormones, TSH may also be one of the key signaling molecules that regulate different brain signals in a male- and female-specific manner. Thyroid-related and neurological disorders exhibit sex-specific differences in prevalence [42]. The reasons for these differences are complex and may include differences in brain structure, function, and stress response, as well as differences in reproductive hormone exposure, social expectations, and experience [43]. However, the molecular mechanisms behind this have not been clarified. Our findings contribute to the understanding of the role of biological sex, physiological, psychological and metabolic factors in mediating the correlation between depressive/suicidal symptoms and ATF. Our results suggest that further studies are needed to explore these potentially relevant risk factors that may influence clinical outcomes.

### Limitations

Our study has several limitations. Firstly, due to the cross-sectional design, we were unable to explore a direct causal relationship between MDD and suicide attempts. Secondly, our participants were admitted to only one psychiatric hospital. In our study, only 24 patients were in the non-ATF subgroup in MDD patients with comorbid suicide attempts. Hence future multicenter and large sample studies are necessary. Thirdly, our sample included only MDD patients, not all depressed patients. Therefore, the results of this study cannot be extended to all depressed patients. Fourthly, the definition of TSH in this study was broad, so our results should be explained with caution in patients with severe thyroid dysfunction. At the same time, the HAMA and PANSS scales used in our study cannot be used to diagnose anxiety and psychiatric disorders and can only quantify symptoms. Finally, we did not use a structured assessment tool to diagnose suicide attempts. In future research, we will cover the methodological limitations of this study with more convincing tools. However, the purpose of our study was to preliminarily investigate patients with abnormal thyroid function in MDD concomitant suicide. Studies on subclinical hypothyroidism and hypothyroidism need to be further explored in the future. Despite these limitations, understanding gender differences in ATF in MDD patients with comorbid suicide attempts may help to optimize antidepressant medication according to biological sex.

## Conclusions

In conclusion, one of the main novel findings of this study was that there were no gender differences in the prevalence of ATF in MDD patients with comorbid suicide attempts. Our study also found that SBP was higher in female FEND MDD patients than in male patients. Moreover, HAMD score, SBP and fasting glucose levels predicted ATF in both male and female MDD patients with comorbid suicide attempts, while severe anxiety, psychotic symptoms and higher TPOAb level were correlated with ATF in female MDD patients, but not in male MDD patients. A longitudinal observation of the HAMD, SBP and glucose concentration in both male and female MDD with comorbid suicide attempts will provide the necessary help to prevent thyroid dysfunction. Our study focused on the thyroid dysfunction in male and female MDD with comorbid suicide attempts in the Chinese Han population to minimize the potential confounding influences. Therefore, for both male and female, our results are more likely to be meaningful in clinical practice. It may be useful to predict the severity of thyroid function in MDD with comorbid suicide attempts, and enables to the conduction of proper evidence-based clinical prevention or intervention.

## Data Availability

The data used and analyzed during the current study are available from the corresponding author.

## Abbreviations

MDD: Major depressive disorder
FT3: Free triiodothyronine
FT4: Free thyroxine
TSH: Thyrotropin
ATF: Abnormal thyroid function
FEDN: First-episode drug-naïve
DSM-IV: Diagnostic and Statistical Manual of Mental Disorders, 4th edition
IRB: Institutional Review Board
HAMA: Hamilton Anxiety Rating Scale
HAMD: Hamilton Depression Rating Scale
PANSS: Positive and Negative Syndrome Scale
TgAb: Anti-thyroglobulin
TPOAb: Thyroid peroxide antibody
ANOVA: One-way analysis of variance
VIF: Variance inflation factors
AUC ROC: Receiver Operating Characteristics
SBP: Systolic blood pressure
